# Cognitive-Affective Predictors of Exertion-Related Fatigue in Primary Biliary Cholangitis: An Experimental Substudy of the SOMA.LIV Project

**DOI:** 10.2147/PRBM.S619844

**Published:** 2026-09-11

**Authors:** Paul Hüsing, Laura Buck, Sabrina Helmke, Johannes Hartl, Bernd Löwe, Christoph Schramm, Anne Toussaint

**Affiliations:** 1 Department of Psychosomatic Medicine and Psychotherapy, University Medical Center Hamburg-Eppendorf, Hamburg, Germany; 2 I. Department of Medicine, University Medical Center Hamburg-Eppendorf, Hamburg, Germany; 3 Hamburg Center for Translational Immunology (HCTI), University Medical Center Hamburg-Eppendorf, Hamburg, Germany; 4 Martin Zeitz Centre for Rare Diseases, University Medical Center Hamburg-Eppendorf, Hamburg, Germany

**Keywords:** primary biliary cholangitis, fatigue, stair-climbing task, fear of movement, kinesiophobia, heart rate variability

## Abstract

**Purpose:**

Primary biliary cholangitis (PBC) is a chronic autoimmune liver disease frequently accompanied by severe fatigue. This exploratory and feasibility SOMA.LIV substudy examined associations between subjective fatigue, psychological factors, heart rate variability (HRV), and physical performance during a brief stair-climbing task in PBC. Fatigue in this study refers to self-reported fatigue, with a focus on subjective post-exertional and post-recovery fatigue in relation to baseline disease-related fatigue.

**Patients and Methods:**

The analysis included 42 participants with PBC (93% female; mean age 56 years) who performed a stair-climbing task with continuous heart rate and HRV monitoring. Self-reports assessed fatigue (pre-, post-, post-recovery), anticipated fatigue, and fear of movement. HRV indices were measured at rest and recovery. Correlational and regression analyses identified predictors of fatigue and task performance.

**Results:**

Psychological factors and HRV were unrelated to performance. Anticipated fatigue strongly predicted post-task fatigue (*β* = 0.69, *p* <0.001), while post-task fatigue predicted fatigue after recovery (*β* = 0.61, *p* <0.001). HRV indices showed no association with fatigue.

**Conclusion:**

Findings suggest that fatigue responses to brief physical exertion in individuals with PBC are driven partly by cognitive–affective expectations, particularly anticipated fatigue, while fear of movement showed only a trend-level association. These results should be interpreted in light of the study’s limitations, including sample size and methodological constraints. Future studies should use larger samples and more demanding exertion paradigms to further elucidate mechanisms underlying exertion-related fatigue in PBC.

## Introduction

Primary biliary cholangitis (PBC) is a chronic autoimmune inflammatory liver disease that primarily affects the small intrahepatic bile ducts and can progress to cirrhosis and liver failure. Its exact pathogenesis remains unclear but is thought to reflect an interplay between genetic predisposition and environmental factors.^1^,^2^ The global prevalence of PBC is increasing, with clusters in northern Europe and the USA. A recent meta-analysis reported a point prevalence of 22.3 cases per 100,000 inhabitants and an incidence of 1.9 per 100,000 in Europe.^3^ Women account for about 90% of cases, with most diagnoses occurring after the age of 40.^2^

PBC presents with a range of symptoms such as pruritus and non-specific upper abdominal discomfort, but fatigue is the most prevalent and debilitating symptom, affecting over half of patients.^2^,^4^ Fatigue is characterized by persistent, overwhelming exhaustion that impairs both physical and cognitive functioning and is not alleviated by rest. Importantly, fatigue is largely refractory to ursodeoxycholic acid (UDCA) therapy, which, while effective in slowing disease progression, does not alleviate this symptom.^5^ Approximately 20% of patients experience severe fatigue, which significantly diminishes quality of life.^6^ Despite research into biological contributors, including cholestasis-related central nervous system changes and elevated inflammatory markers, the pathophysiology of fatigue in people with PBC remains poorly understood.^7^,^8^ Importantly, fatigue is increasingly understood as a multidimensional and heterogeneous construct, such that individuals with comparable self-reported fatigue severity may differ in the underlying biological and psychological processes contributing to their symptom experience. Fatigue may encompass physical, cognitive, and subjective dimensions that do not necessarily reflect identical underlying mechanisms across individuals. In the present study, fatigue was operationalized as self-reported fatigue assessed at baseline and in relation to the exertion task, consistent with the cognitive-affective focus of this exploratory substudy.

Psychological factors have also been implicated in fatigue severity among people with PBC. Constructs such as depression, anxiety, illness perception, cognitive deficits, and reduced self-efficacy have shown associations with fatigue intensity.^7^,^9^ However, psychosocial mechanisms specific to fatigue in PBC are still poorly understood.^10^ A more nuanced understanding of the biopsychosocial mechanisms underlying fatigue is essential for developing effective interventions, particularly with regard to processes related to physical activity. Evidence from other chronic inflammatory conditions indicates that higher levels of physical activity are consistently associated with lower fatigue, independent of disease activity, underscoring the relevance of behavioral and psychological factors in fatigue management.^11^,^12^ Insights from related conditions, particularly chronic fatigue syndrome (ME/CFS), may therefore be informative for understanding fatigue in PBC. In ME/CFS, psychological constructs such as symptom expectation, kinesiophobia (excessive fear of movement due to anticipated fatigue), and catastrophizing (tendency to magnify the threat and negative consequences of symptoms) have been shown to influence fatigue experience and physical performance.^13^,^14^ Symptom expectations serve as internal priors that shape the perception of subsequent experiences,^15^ affecting both subjective fatigue ratings and objective performance.^16–20^ Kinesiophobia can be understood within the framework of the fear-avoidance model, which proposes that fear of movement leads to avoidance of physical activity, thereby contributing to physical deconditioning and the maintenance or exacerbation of fatigue.^21–24^ Experimental stair-climbing studies in people with ME/CFS demonstrated that higher levels of kinesiophobia and catastrophizing are related to poorer performance, although results vary depending on the measure, with anticipated fatigue sometimes predicting longer task duration.^14^ Despite extensive study in ME/CFS, these psychological constructs have not yet been investigated in fatigued people with PBC, even though they may be relevant.

From a biopsychosocial perspective, autonomic nervous system (ANS) dysfunction is increasingly recognized as contributing to fatigue.^25^,^26^ Given that PBC predominantly affects women, sex-related factors may also contribute to variability in symptom presentation and fatigue-related mechanisms. In studies of people with PBC, women have been found to report significantly higher levels of fatigue than age- and disease-duration matched men, a difference that appears to be linked to greater autonomic dysfunction in female patients, even when daytime sleepiness and depressive symptoms are accounted for.^27^ These findings suggest that sex may represent an important source of variability in fatigue-related processes in PBC.

Heart rate variability (HRV) analysis is a non-invasive method to assess ANS activity, capturing dynamic cardiac autonomic changes during resting, active, and recovery states.^28^,^29^ Reduced HRV has been associated with fatigue severity in ME/CFS, and autonomic dysfunction frequently observed across all stages of PBC.^30^ In fatigued people with PBC, HRV is lower compared to non-fatigued individuals, suggesting a link between autonomic abnormalities and fatigue severity independent of disease progression.^30^ Examining HRV during experimental tasks, such as stair-climbing, requires distinguishing tonic (resting) from phasic (reactivity and recovery) measures, with higher resting HRV and greater task-related changes reflecting better cardiac vagal control.^29^

This experimental substudy within the SOMA.LIV project aimed to explore associations between anticipated fatigue, kinesiophobia, autonomic function, and performance during a stair-climbing task in people with PBC. We hypothesized that self-assessed fatigue after the task would correlate with performance measures, and that kinesiophobia would be associated with both fatigue experience and physical performance. Furthermore, we expected that anticipated fatigue, along with demographic variables and baseline fatigue, would predict fatigue experienced after the task and following recovery. Finally, we hypothesized that higher fatigue levels would be associated with reduced cardiac vagal regulation, reflected in lower HRV, and that these autonomic alterations would correspond to greater post-task fatigue. Overall, this substudy was designed as an exploratory experimental component of SOMA.LIV to assess the feasibility of examining cognitive-affective correlates of fatigue in this rare patient population.

## Material and Methods

### Participants and Procedure

The sample consisted of a subsample of patients with a confirmed diagnosis of PBC (*N* = 239) who participated in the DFG-funded SOMA.LIV study, a prospective single-center cohort study investigating fatigue in people with primary sclerosing cholangitis (PSC) and PBC.^10^ This study is part of the interdisciplinary research unit SOMACROSS (RU 5211).^31^ Participants were recruited at the YAEL Center for Autoimmune Liver Diseases, University Medical Center Hamburg-Eppendorf (UKE), Germany, and had to meet SOMA.LIV pre-specified inclusion criteria.^10^ Patients with pre-existing heart, knee, or back conditions were excluded from participation from this embedded study part. Our target sample size of approximately 50 aligns with prior studies that detected statistically significant associations between stair-climbing performance, physical parameters and respective psychological constructs in people with ME/CFS.^13^,^14^ The study was approved by the Ethics Committee of the Medical Chamber Hamburg on December 21, 2020 (processing number: 2020–10196-BO-ff). The research was conducted in accordance with the Declaration of Helsinki.^32^ All participants gave written informed consent prior to their participation. After providing informed consent, participants completed a set of questionnaires assessing their current fatigue levels and fatigue-related expectations. They then performed a stair-climbing task with continuous heart rate (HR) monitoring. Following the task, participants were debriefed and received financial compensation for their participation.

### Stair-Climbing Task

The stair-climbing task was conducted under standardized conditions in a non-public stairwell at UKE. Dahlhausen electrocardiogram (ECG) electrodes were placed on the left chest of each participant by study staff. HR was continuously monitored using the EcgMove4 recording device^33^ during all task phases. Stair-climbing duration was recorded in seconds. The task consisted of three phases: During the resting phase, HR was recorded over 5 minutes while participants sat relaxed with legs bent at a 90° angle and palms resting on their thighs. The activity phase involved climbing up and down two floors (80 steps total) at the participant’s own pace without resting. The recovery phase consisted of another 5-minute seated rest in the same position as the initial resting phase.

### Measures

#### Fatigue

Before the stair-climbing task, participants rated their current fatigue level, average fatigue over the past 24 hours, and worst fatigue over the past 24 hours using a Visual Analogue Scale (VAS) from 0 (no fatigue) to 10 (extreme fatigue). Higher scores indicate greater perceived fatigue severity. The mean of these three items was used to represent baseline fatigue level. Following the activity and recovery phases, participants again rated their experienced fatigue on a VAS. A difference score was calculated by subtracting fatigue experienced immediately after stair climbing from anticipated fatigue to quantify the discrepancy between expected and experienced fatigue.

#### Fatigue Expectancy

To assess fatigue expectancy across daily activities, participants rated their expected fatigue levels on a VAS (0–10) for ten common tasks, including stair-climbing, walking, ironing, running, reading, dishwashing, meal preparation, driving, computer work, and gardening, prior to the stair-climbing task.^13^ Higher scores indicate greater anticipated fatigue associated with the respective activity. The expected fatigue rating for the item “Two floors of stair-climbing and descending” was used as an implicit indicator of anticipated fatigue specific to the stair-climbing task. This single-item measure was used as an exploratory indicator of task-specific fatigue expectancy and has not been psychometrically validated as a standalone scale.

#### Kinesiophobia

Fear of movement (kinesiophobia) was assessed using the German version of the Tampa Scale for Kinesiophobia (TSK-GV).^34^ The TSK-GV was assessed at the 12-month SOMA.LIV follow-up and not contemporaneously with the experimental session. It is a self-report questionnaire measuring fear of movement or re-injury linked to beliefs and thoughts about pain and movement. It consists of 11 items rated on a 4-point Likert scale, with total scores ranging from 11 to 44. Higher scores indicate greater fear of movement and avoidance-related beliefs. Although originally developed for patients with musculoskeletal pain, the TSK has been validated for use across a range of chronic conditions, including fibromyalgia and other pain-related disorders,^35^,^36^ and has also been adapted for fatigue-related contexts.^37^,^38^ A validated German version is available and was used in the present study.^39^ The TSK has shown good internal consistency (*α* = 0.79) and test-retest reliability (ICC = 0.81),^34^ and the German version was validated in clinical populations (*α* = 0.81).^39^ No universally established clinical cut-off values are available for the TSK-GV in this population.

#### Heart Rate Variability (HRV) Analysis

HRV was analyzed following established guidelines.^28^,^29^ Artifacts in ECG recordings were identified and manually corrected. Both time-domain and frequency-domain HRV parameters were extracted to investigate relationships between autonomic function and fatigue measures. Time-domain analyses quantify variability of R–R intervals by assessing dispersion around the mean, while frequency-domain analyses evaluate the power of different frequency components of the heart rate signal.^40^ Given the brief activity phase (< 2 minutes) and the short recording at baseline and recovery (5 minutes), we used the time-domain parameter root mean square of successive differences (RMSSD) and the frequency-based parameter high frequency power (HF), as suggested in previous studies.^41^ Higher RMSSD and HF values are generally interpreted as reflecting greater parasympathetic (vagal) modulation and autonomic flexibility. HRV analyses were conducted using available recordings. Recordings with technically insufficient and incomplete data after visual inspection and manual artefact correction were excluded. No imputation of missing HRV data was performed.

#### Task Performance

Task performance was assessed using heart rate (HR) - a sensitive indicator of psychophysical state influenced by mental and physical stimuli - and task duration in seconds.^42^,^43^ Shorter task duration indicates better physical performance, whereas heart rate responses provide information on physiological demands during task performance.

### Statistical Analysis

Statistical analyses were performed using SPSS version 25,^44^ with a significance level set at *α* = 0.05. Spearman correlation analyses were conducted to examine associations between fatigue-related, psychological, autonomic, and performance-related variables. Correlations among the variables included in the regression models were additionally examined to assess potential associations among predictors. Multiple linear regressions were used to analyze predictors for fatigue severity after the activity phase and after the recovery phase, and predictors of stair-climbing duration. Potential predictors for post-task fatigue included baseline fatigue, anticipated fatigue, kinesiophobia, and RMSSD (time-domain) as HRV parameter at baseline. Potential predictors for fatigue after the recovery phase included anticipated fatigue, post-task fatigue, kinesiophobia, and RMSSD during the recovery phase. For the prediction of stair-climbing duration, the same set of predictors used for post-task fatigue was applied. Patient-reported outcomes for kinesiophobia (TSK-GV) were taken from the 12-month follow-up of the SOMA.LIV study, as these measurements were closest to the timing of the stair-climbing experiment.^10^ Measurement artifacts and outliers for HRV data were manually identified and corrected.

## Results

### Sample Characteristics

Of the 59 participants who completed the study protocol, 42 had complete and analyzable HRV recordings and were included in the final analyses, while 17 participants were excluded due to technically insufficient and incomplete HRV recordings. The final study sample comprised 42 people with PBC, predominantly female (92.9%), with a mean age of 55.86 years (*SD* = 9.95) and a mean body mass index of 26.5 kg/m^2^ (*SD* = 4.81). The mean score for kinesiophobia (TSK-GV) was *M* = 18.76 (*SD* = 7.14). Participants completed the stair-climbing task in an average of 67.1 seconds (*SD* = 10.75). Anticipated fatigue for the stair-climbing task had a mean of *M* = 2.95 (*SD* = 2.04). Descriptive statistics for fatigue, task performance, HR, and HRV are summarized in Table 1.

**Table 1:** Sample Characteristics (N = 42)

Descriptive Variable	*M* ± *SD*
Age	55.86 (9.95)
Female sex (%)	39 (92.9%)
BMI (kg/m^2^)	26.5 (4.81)
Baseline fatigue (0–10)	4.79 (2.16)
Anticipated fatigue for the stair-climbing task (0–10)	2.95 (2.04)
Experienced fatigue after stair climbing (0–10)	2.17 (2.2)
Experienced fatigue after recovery phase (0–10)	2.02 (2.21)
Duration of stair-climbing (in seconds)	67.14 (10.75)
Kinesiophobia (TSK-GV)	18.76 (7.14)
Heart rate at resting phase (bpm)	77.99 (11.31)
Heart rate at activity phase (bpm)	117.05 (12.55)
Heart rate at recovery phase (bpm)	78.91 (13.28)
HRV before stair-climbing task (RMSSD; ms)	19.67 (11.3)
HRV before stair-climbing task (HF; ms^2^)	274.26 (411.32)
HRV after stair-climbing task (RMSSD; ms)	25.37 (15.05)
HRV after stair-climbing task (HF; ms^2^)	419.22 (411.11)

**Abbreviations**: BMI, body mass index; TSK-GV, Tampa Scale of Kinesiophobia – German version; bpm, beats per minute; ms, milliseconds; HRV, heart rate variability; HF, high frequency; RMSSD, root mean square of successive differences.

### Correlation Analyses

Spearman correlation analyses showed that task performance parameters (duration of stair climbing, HR and HR increase during task) were not significantly associated with anticipated fatigue, kinesiophobia, or baseline HRV parameters (see Table 2). Anticipated fatigue was significantly correlated with post-task fatigue (*r* =0.647, *p* <0.001) and baseline fatigue (*r* =0.418, *p* =0.006). Baseline fatigue was also significantly associated with post-task fatigue (*r* =0.519, *p* <0.001).

**Table 2:** Spearman Correlation Matrix of Study Variables and Predictors Included in the Regression Analyses (N = 42)

	Anticipated Fatigue	Baseline Fatigue	Post-Task Fatigue	Δ Fatigue (Anticipated–Post-Task)	Kinesiophobia (TSK-GV)	Baseline HRV (RMSSD)	Baseline HRV (HF)	Recovery HRV (RMSSD)	Recovery HRV (HF)	Task Duration	HR during Task	HR Increase
Anticipated fatigue	1	0.418**	0.647**	0.279	0.102	0.069	−0.085	−0.115	−0.189	0.137	0.002	0.062
Baseline Fatigue		1	0.519**	−0.084	0.381*	−0.110	0.012	−0.180	−0.096	−0.014	0.129	0.049
Post-task fatigue			1	−0.484**	0.363*	−0.073	−0.130	−0.125	−0.112	0.133	−0.014	0.005
Δ Fatigue (anticipated–post-task)				1	−0.316*	0.180	0.135	0.015	−0.052	−0.001	0.108	0.153
Kinesiophobia (TSK-GV)					1	−0.325*	−0.247	−0.367*	−0.311*	0.103	0.243	0.097
Baseline HRV (RMSSD)						1	−0.168	0.699**	0.606**	−0.160	−0.255	0.405**
Baseline HRV (HF)							1	0.055	0.074	0.086	−0.028	0.043
Recovery HRV (RMSSD)								1	0.937**	−0.277	−0.245	0.377*
Recovery HRV (HF)									1	−0.331*	−0.155	0.405**
Task duration										1	−0.009	−0.160
HR during task											1	0.580**
HR increase												1

**Note**: Bold values indicate statistical significance, * = *p* <0.05, ** = *p* <0.01.

**Abbreviations**: TSK-GV, Tampa Scale of Kinesiophobia – German Version; HRV, heart rate variability; RMSSD, root mean square of successive differences; HF = high frequency.

### Multiple Linear Regression Analyses

The multiple linear regression model predicting fatigue severity after the activity phase demonstrated a high goodness-of-fit, with R^2^ = 0.6 and adjusted R^2^ = 0.56, consistent with Cohen’s (1988) guidelines.^45^ The overall model was statistically significant, *F*(4, 37) = 13.883, *p* <0.001. Although the combination of predictors significantly predicted post-stair-climbing fatigue, anticipated fatigue was the only individual predictor reaching statistical significance (*t* = 5.919, *p* <0.001; see Table 3). In a supplementary regression analysis excluding anticipated fatigue, baseline fatigue was significantly associated with post-task fatigue (*β* = 0.412, *p* =0.011), while kinesiophobia and HRV were not significant predictors. The model explained 22.2% of the variance (adjusted R^2^ =0.160), compared with 60.0% in the model including anticipated fatigue (Supplementary Table 1).

**Table 3:** Multiple Linear Regression Analyses Predicting Experienced Fatigue After Stair-Climbing and After Recovery Phase

Variable	Dependent variable: Fatigue After Stair-Climbing					Dependent Variable: Fatigue After Recovery Phase				
	B (unstand ardized) (CI 95%)	Standard Error	*β* (standardized) (CI 95%)	T	Significance (*p*)	B (unstand ardized) (CI 95%)	Standard Error	*β* (standardized)	T	Significance (*p*)
Anticipated fatigue	0.743 (0.489; 0.998)	0.126	0.689	5.919	**<0.001**	0.059 (−0.298; 0.416)	0.176	0.054	0.334	0.740
Baseline fatigue	0.082 (−0.176; 0.341)	0.128	0.081	0.645	0.523					
Fatigue after task						0.618 (0.277; 0.958)	0.168	0.613	3.678	**<0.001**
Kinesiophobia	0.047 (−0.028; 0.122)	0.037	0.153	1.271	0.212	0.074 (−0.001; 0.148)	0.037	0.237	2.01	0.052
HRV baseline (RMSSD)	−0.018 (−0.062; 0.026)	0.022	−0.093	−0.84	0.406					
HRV recovery (RMSSD)						−0.007 (−0.041; 0.026)	0.017	−0.05	−0.445	0.659
	Overall model		*p*	*R*^2^	Adjusted *R*^2^	Overall model		*p*	*R*^2^	Adjusted *R*^2^
	F (4,37) = 13.883		**<0.001**	0.6	0.557	F (4, 37) = 12.791		**<0.001**	0.58	0.535

**Note**: Bold values indicate statistical significance.

**Abbreviations**: HRV, Heart rate variability; RMSSD, Root Mean Square of Successive Differences.

For fatigue after the recovery phase, the regression model was also significant, *F*(4,37) = 12.791, *p* <0.001, with R^2^ = 0.58 (adjusted R^2^ = 0.535), indicating a high goodness-of-fit. In this model, fatigue after recovery was significantly associated with post-task fatigue (*t* = 3.678, *p* <0.001), whereas the association with kinesiophobia did not reach statistical significance (*p* =0.052).

Regarding the duration of the stair-climbing task, the regression model was not significant, *F*(4,37) = 1.302, *p* =0.287, with an adjusted R^2^ = 0.029. None of the included predictors reached statistical significance for predicting task duration.

## Discussion

This study provides preliminary evidence that expectations are associated with fatigue experiences in people with PBC during low-intensity physical activity. While none of the psychological variables significantly predicted objective task performance (ie, stair-climbing duration or HR), anticipated fatigue emerged as the only significant predictor of fatigue following the task. This finding aligns with previous research in people with ME/CFS,^13^ supporting the notion that anticipatory cognitions about fatigue can influence subjective symptom perception, even in the absence of measurable impairments in physical performance. However, given that both anticipated and post-task fatigue were assessed subjectively and within a short time interval, this association should not be interpreted as evidence of a causal effect of expectations on subsequent fatigue experience. Anticipated fatigue may partly reflect individuals’ appraisal of their expected symptom response, which could overlap with subsequent subjective fatigue ratings. Evidence from nocebo research further highlights that negative symptom expectations may manifest as catastrophizing thoughts and negative affect, thereby intensifying avoidance behavior and increasing psychological distress.^23^ Importantly, while early placebo studies demonstrate that verbal suggestions can reduce fatigue and enhance physical performance,^46^ emerging nocebo findings indicate that negative expectations can exacerbate self-reported exhaustion,^47^ impair task performance,^48^ and increase the urge to disengage from cognitively demanding activities in healthy individuals.^49^ A recent study in people with PBC demonstrated that verbal instructions aiming to shape fatigue expectations influenced the motivational urge to stop a cognitive task, with fatigue-inducing instructions leading to greater disengagement compared to fatigue-reducing instructions, even though both groups reported similar levels of subjective fatigue.^50^ Together, these observations support the interpretation that anticipated fatigue is an important correlate of subjective fatigue experience and activity engagement across different clinical and experimental contexts. In contrast to studies in people with ME/CFS, where anticipated symptoms often exceed actual physiological responses, in people with PBC this pattern may indicate a distinct cognitive-affective profile, with fear-based beliefs rather than distorted symptom expectations driving sustained fatigue. Such disease-specific differences highlight the importance of tailored psychological models to understand fatigue across conditions.

Beyond immediate task-related fatigue, our findings suggest that fatigue persistence after recovery is primarily associated with post-task fatigue, while kinesiophobia showed a non-significant association with fatigue after recovery. Notably, participants reported overall moderate levels of fatigue already at baseline, indicating that these effects occurred in the context of generally non-severe fatigue. Given the limited sample size and exploratory nature of the analyses, this finding should be considered inconclusive and interpreted cautiously rather than as evidence for a meaningful association. Nevertheless, movement-related fear may still represent a potentially relevant mechanism warranting further investigation in larger studies. Conceptually, fear of movement may modulate the interpretation of bodily sensations during recovery, leading to sustained perceptions of exhaustion even after low-intensity exertion. This mechanism could reflect heightened vigilance or negative appraisal of interoceptive signals among individuals with greater kinesiophobia.

Regarding physiological measures, baseline HRV data confirmed previously reported reductions in vagal tone in people with PBC.^30^ However, the absence of associations between HRV indices and fatigue severity may stem from methodological factors. The brief duration of the stair-climbing task (mean ≈ 67 seconds) likely limited autonomic reactivity, while characteristics of the recovery phase may have influenced HRV, obscuring dynamic changes. Furthermore, technical issues with HRV recordings resulted in the exclusion of some participants from HRV analyses, reducing the available sample size and potentially limiting statistical power. Importantly, the absence of observed associations between HRV indices and fatigue outcomes should not be interpreted as evidence against a role of autonomic mechanisms in PBC-related fatigue, but rather as reflecting the methodological constraints and exploratory nature of the present analysis. In addition to these methodological factors, individual variability and sample characteristics may also have contributed to the absence of observed associations. These factors emphasize the need for longer, ecologically valid exertion paradigms and improved sensor reliability to capture meaningful autonomic dynamics in future research.

The relatively low-intensity nature and short duration of the task may also have limited the extent to which post-exertional fatigue increases were elicited. Interestingly, some participants reported higher fatigue before than after the task, which may reflect anticipatory responses or day-to-day symptom variability rather than sustained exertional effects. Compared with stair-climbing paradigms in people with ME/CFS, the present task was both shorter and perceived as less demanding, potentially attenuating fatigue-related physiological responses. Nonetheless, the study successfully demonstrated that psychological factors, especially anticipated fatigue, and potentially kinesiophobia, are associated with subjective fatigue experiences, even in the absence of effects on physical performance. From a clinical perspective, this underscores the relevance of addressing expectation and fear-based processes in PBC-related fatigue. Cognitive-behavioral and psychoeducational interventions targeting maladaptive fatigue expectations or movement-related fears may help reduce perceived fatigue and improve activity engagement. Aligning anticipated and actual exertion could further mitigate avoidance behaviors and enhance daily functioning.

Methodologically, the study’s strengths include its well-controlled design, integration of psychological and physiological variables, and application of a validated stair-climbing paradigm in a population where fatigue mechanisms remain underexplored. However, several limitations should be acknowledged. The relatively small sample size (*N* = 42) in relation to the number of predictors limits the robustness of the regression analyses and may have reduced the ability to detect smaller effects. Therefore, non-significant findings should not be interpreted as evidence of absence. The brief and low-intensity nature of the physical task may have limited the extent to which exertional fatigue responses were elicited, potentially emphasizing cognitive-affective aspects of fatigue and limiting generalizability to real-world fatigue experiences. Furthermore, the findings should be interpreted in light of the specific sample and study context, including the predominantly female sample, which may limit generalizability to men with PBC and other populations or settings. The TSK-GV was originally developed to assess pain-related fear and, although adapted for fatigue-related contexts, has not been specifically validated in people with PBC. Findings involving this measure should thus be interpreted with caution. Moreover, kinesiophobia was assessed at the 12-month SOMA.LIV follow-up rather than immediately before the experimental task, which may have introduced additional measurement variability and should be considered when interpreting the exploratory associations involving kinesiophobia.

The absence of a control group further constrains interpretability and single-item VAS ratings capture only a limited aspect of fatigue’s multidimensional nature. While the single-item VAS allowed assessment of task-specific anticipated fatigue with minimal participant burden, future studies may benefit from incorporating multidimensional fatigue instruments, such as the Multidimensional Fatigue Inventory (MFI), to more comprehensively capture the complexity of fatigue. Thus, the present findings should be considered as preliminary evidence supporting the feasibility and relevance of investigating cognitive-affective contributors to exertion-related fatigue in PBC, rather than as establishing definitive mechanisms.

Future research should employ more challenging or ecologically valid physical tasks, possibly in patients with higher baseline fatigue or in the context of treatment studies, to explore fatigue trajectories and interoceptive accuracy under more demanding conditions. In addition, cognitive fatigue paradigms or combined physical and cognitive tasks may help to further elucidate fatigue-related mechanisms across different domains. Comparative studies in people with ME/CFS and other populations with chronic illness could clarify whether observed mechanisms are disease-specific or represent generalizable cognitive-affective processes underlying fatigue. Future studies using larger and more diverse samples and incorporating neurobiological approaches may help further clarify the mechanisms underlying subjective fatigue experiences and expectation-related fatigue responses. Future research should further examine the role of sex-related heterogeneity in fatigue mechanisms, particularly given previous evidence of sex differences in fatigue severity and autonomic regulation in PBC. Despite these limitations, the present study provides valuable preliminary evidence that expectation- and fear-related cognitions contribute to fatigue perception in PBC, offering a foundation for future experimental and therapeutic investigations in this under-researched patient group.

## Conclusion

In summary, this experimental substudy provides preliminary evidence that cognitive-affective factors, particularly anticipated fatigue, are associated with subjective fatigue experiences during low-intensity physical activity in individuals with PBC. While no associations were observed between psychological variables and objective performance or autonomic measures, anticipatory cognitions emerged as the most robust predictor of post-task fatigue. These findings highlight the relevance of considering cognitive-affective processes when investigating fatigue experiences in PBC, while acknowledging that subjective fatigue may arise from heterogeneous underlying mechanisms across individuals. Future research using larger samples, more ecologically valid paradigms, and approaches capable of characterizing biological variability is needed to further clarify the mechanisms underlying expectation-related fatigue responses.

## Data Availability

The data that support the findings of this study are available from the corresponding author upon reasonable request.
